# Supplementary material for: Exposure to e-cigarette TV advertisements among U.S. youth and adults, 2013–2019
Source: PLoS One. 2021 May 7;16(5):e0251203. doi: 10.1371/journal.pone.0251203 (PMC8104405; doi:10.1371/journal.pone.0251203)
Supplement: S1 Table — (DOCX) [file pone.0251203.s001.docx]

**S1 Table. List of keywords to retrieve e-cigarette TV advertising data from Kantar Media.**

| **Generic terms**  electronic cigarette  e-cig  ecig  tobacco shop  **slang terms**  vape  vapor  vaping  **Components**  e-liquid  e-juice  cartridge  pod | **Product brands**  21st Century Smoke  24/7  AirVape  Alien Visions  American Blue Tip  American Smoke  Anarchist  Apollo  Aspire  Atlantic  Avanti  Bedford Slims  Big Bang  Black Box  Blackcat  Blackout  Blaze  Bloog  Blu Cigs  BocaVapes  Bodyshot  Boulder  Breathe  Breez  Buck Naked  Bull Smoke  Bulldog  Caterpillar Vapes  Centurion  Charleston  Choose Smoke Free  Cig2o  Cigalectric  Cigalex  Cigana  Cigavette  CigFree  Cigirex  Cigr8  CigReds  Citizen  Clayton  Clear Vapors  Clearette  Craving Vapor  Crown Lion  Crown Mist  Crown Seven  Cuttwood Boss  Dab R  Direct  D-Lite  Dragonfly  Durasmoke  E Cig 101  ECBlend  ECigAir  ECigared  Eco-Cigs  Eko  ElectronicStix  Elektro  E-Lites  Eluma  E-Luminate  Emerald Lux  Emperor  Envy  EON  Eonsmoke  ePuffer  EsmokeUSA  Esvape  E-Swisher  Eversmoke  Evolution  Ewize Smoker  EZ Cig  Fifty-One  Fin  Finiti  Flair Xtreme  FLUID  Fort Myers  Freedom  Fuma  Gamucci Micro  Gatorvapor  Ghost MV1  Glamsmoke  GMK  Go Pen Plus  Good Vapes  Great Lakes  Green Earth  Green Machine  Green Nicotine  Green Smoke  Greensmartliving  Greentip  HempVap  Hi-Tech  Honey Stick  Hustler  Imperial Smoke  Inno-Vapor  Instead  Intellicig  Invisi-Vapor  Ismoke  Jak  Jasper  Joyetech  Juicycigs  JUUL  Kangertech  Karma  Kilo  Knight  Koopor  Krave It  KURE  Leap Go  LeCig  LEX12  Liberro  Liberty  LifeCig  Lipmist  Lizard  Logic  Luci  Lux  MaCoca  MadVapes  Magic  MarkTen  Maxx  Metro | Mimic  Mistic  Mod  Monster Vapin  Neo  Never Light Again  New Day  NEwhere  Nicotek  Nic-Out  Nicstick  Njoy  No 7  North American  NoSmoke 300  Nulife  NutriCigs  Nuvo  Nu-XCBD  O.Pen  O2Pur  ONE80  ONO  Ovale  Panama  Perfection  Platinum  Plumes  Power  Prado  Prosmoke  ProVape  PUF Cigs  Puff  PuffBar  Pure Cigs  PureSmoke  RAD Vapor  Real Feel  Red Dot Smoke  Rejuve  Renew Cigs  Ripe Vap  RJ Reynolds  Royal Flush  Royale  S2 Vape  Sabor  Saphire  Sapphyre  Seminole  Shamrock  SKYCIG  SmartFixx  Smok  Smoke  Smokeless  SMOKER FRIENDLY  Smokers 1 Choice  Smokers Ally  SmokeTip  Smoking Everywhere  Smoking Stick  Smoking Without Smoke  Smooth Smoking  SMOX DR  Solar Cigarette  Solaris  SoundVape  South Beach Smoke  Spark Industries  Sparki  Splendid Smoke  Square  Starfire  Stems  Sunrise Vapor  Swisher  TKO Vapor  Totally Wicked  Triple Crown  TRYST  Urus Cigs  US Vapor  V2 Cigs  Vantage Smoke  Vapage  Vape A Storm  Vape It  Vape King  Vapers Corner  Vapir Prima  Vapor Couture  Vapor Daze Decades  Vapor Diamond  Vapor Envy  Vapor Jadore  Vapor Mate  Vapor Station  Vapor Tobacco  Vapor Vortex  Vapor Wise  Vapor Wize  Vapor4You  VaporandCo  VaporBurst  VaporCorp  Vaporetti  Vaporfection  Vaporfection  VaporFi  Vaporin  Vaporium  VaporKing  VaporNine  Vaporsmarts  VaporX  Vaporz  Vapure  VChic  Vero  VESSEL  Vibe  Victor  Victory  Viola Vapes  VMR Products  Volcano  Volt  Voodoo  Vuse  Vype  White Cloud  White Horse  Wild West  Wow Vapor  X2O Kronos  ZeroCig  Zoom |
| --- | --- | --- |
