# Supplementary material for: Exposure to e-cigarette TV advertisements among U.S. youth and adults, 2013–2019
Source: PLoS One. 2021 May 7;16(5):e0251203. doi: 10.1371/journal.pone.0251203 (PMC8104405; doi:10.1371/journal.pone.0251203)
Supplement: S2 Table — (DOCX) [file pone.0251203.s002.docx]

**S2 Table. Total quarterly e-cigarette TV advertising expenditure and quarterly e-cigarette TV ratings overall and by age groups, 2013 to 2019.**

| **Time** | **Youth (12-17) TRPs** | **Adult (18+) TRPs** | **Household GRPs** | **Expenditure**  **(in 1000 US dollar)** |
| --- | --- | --- | --- | --- |
| **2013 Q1** | 172.4 | 385.3 | 697.6 | 3,867.4 |
| **2013 Q2** | 352.6 | 725.3 | 1,308.9 | 7,840.2 |
| **2013 Q3** | 256.2 | 446.1 | 819.9 | 4,324.9 |
| **2013 Q4** | 248.0 | 435.5 | 791.3 | 5,377.3 |
| **2014 Q1** | 294.5 | 564.8 | 998.1 | 9,937.4 |
| **2014 Q2** | 159.7 | 230.4 | 423.8 | 3,736.6 |
| **2014 Q3** | 260.4 | 581.4 | 1,032.9 | 8,620.1 |
| **2014 Q4** | 182.4 | 311.2 | 546.2 | 5,637.3 |
| **2015 Q1** | 250.2 | 522.6 | 911.7 | 7,582.0 |
| **2015 Q2** | 241.2 | 563.0 | 1,011.0 | 5,712.4 |
| **2015 Q3** | 0.0 | 0.0 | 0.0 | 466.7 |
| **2015 Q4** | 237.4 | 589.4 | 1,048.6 | 6,525.4 |
| **2016 Q1** | 163.7 | 388.5 | 677.7 | 4,486.9 |
| **2016 Q2** | 89.1 | 201.7 | 356.0 | 3,944.9 |
| **2016 Q3** | 115.7 | 424.2 | 761.3 | 4,826.3 |
| **2016 Q4** | 118.7 | 415.4 | 740.4 | 5,545.4 |
| **2017 Q1** | 166.5 | 661.9 | 1,160.6 | 5,865.0 |
| **2017 Q2** | 2.3 | 8.3 | 15.0 | 567.1 |
| **2017 Q3** | 12.0 | 29.3 | 54.0 | 1,606.8 |
| **2017 Q4** | 2.6 | 7.6 | 14.0 | 1,121.7 |
| **2018 Q1** | 0.6 | 2.0 | 3.7 | 1,362.7 |
| **2018 Q2** | 2.4 | 5.1 | 9.3 | 288.0 |
| **2018 Q3** | 0.0 | 0.0 | 0.0 | 211.0 |
| **2018 Q4** | 0.1 | 0.3 | 0.6 | 203.7 |
| **2019 Q1** | 100.8 | 528.1 | 923.6 | 12,120.9 |
| **2019 Q2** | 115.4 | 702.5 | 1,239.0 | 13,665.5 |
| **2019 Q3** | 316.8 | 1,701.9 | 3,015.4 | 31,287.1 |
| **2019 Q4** | 64.9 | 351.6 | 619.1 | 6,531.7 |
